# Outer Plexiform Layer Structures Are Not Altered Following AAV-Mediated Gene Transfer in Healthy Rat Retina

**DOI:** 10.3389/fneur.2017.00059

**Published:** 2017-02-23

**Authors:** Bert C. Giers, Daniela Klein, Alexandra Mendes-Madeira, Carolina Isiegas, Birgit Lorenz, Silke Haverkamp, Knut Stieger

**Affiliations:** ^1^Department of Ophthalmology, Justus-Liebig-University Giessen, Giessen, Germany; ^2^INSERM UMR 1089, University of Nantes, Nantes, France; ^3^Max-Planck-Institute for Brain Research, Frankfurt, Germany; ^4^Institute of Cellular and Molecular Anatomy, Goethe-University Frankfurt, Frankfurt, Germany

**Keywords:** RPE65, gene therapy, retinal degeneration, Leber congenital amaurosis, retinal detachment, synaptic plasticity

## Abstract

Ocular gene therapy approaches have been developed for a variety of different diseases. In particular, clinical gene therapy trials for RPE65 mutations, X-linked retinoschisis, and choroideremia have been conducted at different centers in recent years, showing that adeno-associated virus (AAV)-mediated gene therapy is safe, but limitations exist as to the therapeutic benefit and long-term duration of the treatment. The technique of vector delivery to retinal cells relies on subretinal injection of the vector solution, causing a transient retinal detachment. Although retinal detachments are known to cause remodeling of retinal neuronal structures as well as significant cell loss, the possible effects of this short-term therapeutic retinal detachment on retinal structure and circuitry have not yet been studied in detail. In this study, retinal morphology and apoptotic status were examined in healthy rat retinas following AAV-mediated gene transfer *via* subretinal injection with AAV2/5.CMV.d2GFP or sham injection with fluorescein. Outer plexiform layer (OPL) morphology was assessed by immunohistochemical labeling, laser scanning confocal microscopy, and electron microscopy. The number of synaptic contacts in the OPL was quantified after labeling with structural markers. To assess the apoptotic status, inflammatory and pro-apoptotic markers were tested and TUNEL assay for the detection of apoptotic nuclei was performed. Pre- and postsynaptic structures in the OPL, such as synaptic ribbons or horizontal and bipolar cell processes, did not differ in size or shape in injected versus non-injected areas and control retinas. Absolute numbers of synaptic ribbons were not altered. No signs of relevant gliosis were detected. TUNEL labeling of retinal cells did not vary between injected and non-injected areas, and apoptosis-inducing factor was not delocalized to the nucleus in transduced areas. The neuronal circuits in the OPL of healthy rat retinas undergoing AAV-mediated gene transfer were not altered by the temporary retinal detachment caused by subretinal injection, the presence of viral particles, or the expression of green fluorescent protein as a transgene. This observation likely requires further investigations in the dog model for RPE65 deficiency in order to determine the impact of RPE65 transgene expression on diseased retinas in animals and men.

## Introduction

Adeno-associated virus (AAV)-mediated gene therapy has facilitated tremendous progress in the therapy of hereditary retinal dystrophies in the last decade. A characteristic feature of retinal dystrophies is the progressive degeneration of photoreceptors in the retina leading to severely reduced vision to the point of blindness often early in life (1, 2). A growing number of genetic mutations have been identified over the past decades that can cause retinal dystrophy (3), among which mutations in the gene encoding the retinal pigment epithelial protein of 65 kDa (RPE65) are best characterized (4). This isomerase plays a crucial role in the visual cycle restoring the light sensitive chromophore 11-cis retinal from all-trans retinal (5, 6). Mutations in the RPE65 gene are associated with Leber’s congenital amaurosis type 2 or early onset severe retinal dystrophy, depending on the age of onset of severe visual impairment, which may vary widely in affected individuals (2, 7).

Several animal models have been described to model the pathological findings of RPE65 deficiency in human patients, most importantly the naturally occurring canine model of RPE65 deficiency, the Swedish Briard dog (8). All of these animal models have been successfully used to implement new therapeutic strategies, showing that AAV-mediated gene therapy is able to restore significant vision in affected animals (9–13).

First results from clinical trials were published in 2008, showing that AAV-mediated gene transfer was safe and efficacious in human patients (14–17). Functional improvement was restricted most often to improved light sensitivity and occasionally to visual acuity, but there was no rescue of ERG recordings in treated individuals. Some patients developed extrafoveal fixation points, thereby suggesting preserved plasticity in retinal circuitry (18). Successive clinical trials and long-term follow-up have confirmed the safety of the treatment.

Why the efficacy results of the clinical trials fell short of the promising results of the murine and canine studies remains unclear. Different explanations have been discussed. One important point is that the genetic situation in humans is far more complex and heterogeneous than in the genetically homogeneous mouse lines or pure-bred dogs, thereby resulting in a greater variety in clinical symptoms and disease progression. The expression of non-functional proteins in patients with missense mutations could cause interferences with the therapeutic gene (19). Furthermore, the level of transgene expression seems to be crucial and may not be as high as necessary to restore enzyme function (20). Possible repercussions of these findings on retinal function have yet to be examined. Ultimately, subretinal injection and gene transfer as well as transgene expression could all affect retinal structures. Changes in retinal circuitry and cell function as well as gliosis and apoptosis might impair signaling pathways within the retina.

It has been shown that the synaptic circuitry within the retina retains a certain degree of plasticity and is perceptive to dynamic changes. In non-human primates, it has been demonstrated that change from dichromatic to trichromatic vision was possible following gene transfer of the missing chromophore (21, 22). The formation of ectopic synapses (synaptic sprouting) has been shown in animal models of different retinal degenerative diseases (23–26) as well as in genetically engineered mouse lines with missing rod or cone output (27, 28) and is typically considered to be a marker of ongoing neurodegeneration.

Experimental retinal detachments have also been shown to cause changes in the synaptic architecture of the outer plexiform layer (OPL), expression of photoreceptor-specific proteins and inner and outer segment structure (29–31). Thus, the transient retinal detachment caused by subretinal injections for gene delivery may also cause changes to retinal circuitry. Such effects have, however, not been further examined, owed perhaps to the circumstance that no severe adverse events in the preclinical gene therapy studies were observed. However, the results from the clinical studies revealed a complex image of reaction, both on the morphological and the functional levels suggesting that further understanding of the effects of therapeutic retinal detachment is important. The aim of our study was to examine the effects of subretinal AAV delivery on OPL structures and retinal circuitry in healthy retinas.

## Materials and Methods

### Animals, Vector Preparation, Subretinal Injection, and Tissue Preparation

All animals were cared for in accordance to the ARVO statement for the use of animals in ophthalmic and vision research and EU directive 2010/63/EU. The protocol was approved by the Institutional Animal Care and Use Committee of the University of Nantes.

A total of 12 healthy Sprague-Dawley rats were used in this study. Subretinal injection was performed as previously described (32). Briefly, a transscleral/transchoroidal approach was used by first puncturing the sclera and the choroid. A 33-gauge needle was then inserted into the globe *via* the sclerotomy in a tangential direction under an operating microscope. Fluorescein was added to the viral solution at a 1/1,000 final dilution, and the injection mixture was delivered into the subretinal space. The accuracy of the injections was monitored by fluorescence fundus photography immediately following the injection procedure.

Eight animals received unilateral subretinal injection with either AAV2/5.CMV.d2GFP or sham injection with fluorescein. Two animals were injected subretinally with AAV2/5.CMV.d2GFP in both eyes. Injected volume amounted to 1.5 µL per eye, and vector titer was 1 × 10^11^ vg/ml. Two untreated animals served as untreated controls. Animals were sacrificed 1 month and (in two cases) 12 months after treatment, respectively (Table 1).

**Table 1:** **Animals, age at treatment and examination, vector titer, injected volume, and fixation times**.

Animal	Eye	Treatment	Injected volume (µL)	Titer	Fixation (min)	Age at treatment (months)	Age at examination (months)	Examinations performed				
								Fd	LM	EM	TUNEL	Quant.
R1	OS	–	–	–	10	–	12		X			
	OD	–	–	–	30				X			
R2	OS	–	–	–	10	–	12		X			
	OD	–	–	–	30				X			
R3	OS	AAV2/5.CMV.d2GFP	1.5	3 × 10^11^ vg/ml	10	6	18	X	X			
	OD	AAV2/5.CMV.d2GFP	1.5	3 × 10^11^ vg/ml	30			X	X			
R4	OS	AAV2/5.CMV.d2GFP	1.5	3 × 10^11^ vg/ml	10	6	18	X	X			
	OD	AAV2/5.CMV.d2GFP	1.5	3 × 10^11^ vg/ml	20			X	X			
R5	OS	–	–	–	15	6	7	X	X		X	X
	OD	AAV2/5.CMV.d2GFP	1.5	3 × 10^11^ vg/ml	15			X	X		X	X
R6	OS	–	–	–	15	6	7	X	X		X	X
	OD	AAV2/5.CMV.d2GFP	1.5	3 × 10^11^ vg/ml	15			X	X		X	X
R7	OS	–	–	–	15	6	7	X	X		X	X
	OD	AAV2/5.CMV.d2GFP	1.5	3 × 10^11^ vg/ml	15			X	X		X	X
R8	OS	–	–	–	EM	6	7	X	X	X		
	OD	AAV2/5.CMV.d2GFP	1.5	3 × 10^11^ vg/ml	EM			X	X	X		
R9	OS	–	–	–	EM	6	7	X	X	X		
	OD	AAV2/5.CMV.d2GFP	1.5	3 × 10^11^ vg/ml	EM			X	X	X		
R10	OS	–	–	–	15	6	7	X	X		X	X
	OD	Fluorescein	1.5	1:1,000	15			X	X		X	X
R11	OS	–	–	–	15	6	7	X	X		X	X
	OD	Fluorescein	1.5	1:1,000	15			X	X		X	X
R12	OS	–	–	–	15	6	7	X	X		X	X
	OD	Fluorescein	1.5	1:1,000	15			X	X		X	X

*OD, right eye; OS, left eye; Fd, funduscopy; LM, light microscopy and immunohistochemistry; vg/ml, vector genomes per milliliter; EM, electron microscopy; TUNEL, TUNEL assay; Quant., quantification of outer plexiform layer synapses*.

AAV2/5.CMV.d2GFP was produced in the Vector Core at the University Hospital of Nantes (http://www.vectors.nantes.inserm.fr), and the titer was determined by dot blot and expressed as vector genomes (vg) per milliliter. Viral preparation was manufactured using the HEK293 cell transfection method and purified by cesium chloride density gradients followed by extensive dialysis against phosphate-buffered saline (PBS).

Eyes were enucleated, anterior segments removed, and the eyecups immersion fixed in 4% paraformaldehyde (PFA) in 0.1 M phosphate buffer (PB, pH 7.4) for 10–30 min, depending on the antibodies used (Table 2). After fixation, the retinas were dissected from the eyecup and cryoprotected in graded sucrose solutions (10, 20, and 30%, respectively).

**Table 2:** **Primary antibodies**.

Antibody	Host	Antibody type	Dilution	Specificity in the rat retina	Manufacturer	Batch-No.
Apoptosis-inducing factor	rb	Polyclonal	1:100	N/A	Cell Signaling Technologies, Danvers, MA, USA	4642
Calcium-binding protein (calbindin)	ms	Monoclonal IgG	1:1,000	Horizontal cells and some amacrine and ganglion cells	Swant, Bellinzona, Switzerland	300
Calbindin	rb	Polyclonal	1:2,000	Horizontal cells and some amacrine and ganglion cells	Swant, Bellinzona, Switzerland	CB-38a
C-terminal-binding protein 2 (CtBP2)	rb	Polyclonal	1:5,000	RIBEYE in synaptic ribbons of photoreceptor terminals and bipolar cells, CtBP2 in cell nuclei	Synaptic-Systems, Goettingen, Germany	193-003
Dihydropyridine (DHP)	ms	Monoclonal IgG	1:1,000–1:2,000	Postsynaptic calcium channels in invaginating dendrites of rod bipolar cells and ON cone bipolar cells	Chemicon (Millipore), Billerica, MA, USA	MAB427
Glial fibrillary acidic protein	rb	Polyclonal	1:1,000	Astrocytes, Müller cell endfeet at inner limiting membrane	Chemicon (Millipore), Billerica, MA, USA	AB5804
Green fluorescent protein	rb	Polyclonal IgG	1:2,000	N/A	Invitrogen, San Diego, CA, USA	A-11122
Protein kinase C (PKCα)	rb	Polyclonal	1:10,000	Rod bipolar cells and a subtype of amacrine cell	Sigma-Aldrich, St. Louis, MO, USA	P4334
Postsynaptic density protein of 95 kDa	ms	Monoclonal IgG	1:500	Presynaptic membrane of photoreceptor terminals in OPL, postsynaptic density at AMPA receptors in IPL	Affinity Bioreagents, Golden, CO, USA	MA1-046

*ms, mouse; rb, rabbit; N/A, not applicable; IPL, inner plexiform layer; OPL, outer plexiform layer*.

### Immunohistochemistry and Confocal Microscopy

Immunohistochemistry labeling was performed using the indirect fluorescent method as previously described (33). Tissue was embedded in OCT medium (Jung, Nussloch, Germany), and vertical frozen sections of 16 µm were cut with a Leica cryostat.

Sections were washed in 0.1 M PB for 3 × 10 min and incubated overnight with primary antibodies diluted in 3% normal donkey serum, 1% bovine serum albumin, and 0.5% Triton X-100 in PBS (pH 7.4) at room temperature. Thereafter, sections were washed in PB and incubated with the secondary antibody for 1 h. After a final washing step, cover slips were mounted with Aqua PolyMount (Polysciences, Warrington, PA, USA) and the sections were stored at 4°C for microscopy.

The primary antibodies used are listed in Table 2. As secondary antibodies both donkey anti-mouse and donkey anti-rabbit Alexa-Fluor 488 (green fluorescence; MoBiTech/Invitrogen, Karlsruhe, Germany) and Cy3 (red fluorescence; Dianova, Hamburg, Germany) were used, each diluted 1:500.

Confocal micrographs were taken using a confocal microscope (AxioMOT with LSM 5 Pascal Module, Carl Zeiss, Jena, Germany) equipped with Argon and helium–neon lasers. Images were acquired sequentially for each channel with either a Plan Neofluar 40×/1.30 Oil objective or a Plan-Apochromat 63×/1.40 Oil objective (Carl Zeiss, Jena, Germany) and using the LSM 5 Pascal V3.2 SP2 software (Carl Zeiss, Jena, Germany). Final image adjustment for brightness and contrast was carried out with Photoshop CS5.1 (Adobe Systems, Munich, Germany).

### Electron Microscopy (EM)

Electron microscopy was carried out as previously described (34). Briefly, eyecups were immersion fixed with 2.5% glutaraldehyde and 4% PFA in PB. Subsequently, the retinae were dissected and washed in 0.1 M cacodylate buffer (pH 7.4) and thereafter incubated in 2% osmium tetroxide in cacodylate buffer and dehydrated in graded ethanol solutions (30, 50, 70, 90, and 2 × 100%). For contrasting, 2% uranyl acetate was added at the 70% ethanol dehydration step. After pre-incubation with propylene oxide and epon (1:1) for 30 min, the sample was incubated overnight in pure epon, then embedded in silicone, and dried at 60° for 2 days. Serial ultrathin vertical sections were cut at 60–80 nm using a microtome, collected onto formvar-coated copper grids and then stained with uranyl acetate and lead citrate. Examination was performed with a LEO 912 AB Omega transmission electron microscope (Carl Zeiss, Jena, Germany).

### Quantification of Ribbon Structures

In three animals that received unilateral injection with the green fluorescent protein (GFP) vector and three animals that received unilateral sham injection with fluorescein, the number of synaptic contacts between rod photoreceptors and rod bipolar cells was quantified, the contralateral untreated eyes serving as controls. For this purpose, three representative vertical sections from corresponding areas of each eye were examined. For better comparability, alignment of the samples, magnification, retinal thickness, and number of photoreceptor cell layers were kept constant throughout the examined sections. All sections were double labeled for C-terminal-binding protein 2 (CtBP2) and dihydropyridine (DHP). Acquired micrographs were split into the two respective color channels using ImageJ (Version 1.46i, W. S. Rasband, National Institutes of Health, Bethesda, CA, USA), and all clearly discernible synaptic ribbons in rod terminals and DHP-positive dendritic tips of rod bipolar cells were counted by the same examiner over the whole width of the microscopic scan (146 µm) using the Cell Counter PlugIn (Kurt de Vos, Academic Neurology, University of Sheffield, UK). Statistical analysis was performed with SPSS version 21 (IBM, Ehningen, Germany), using a one-way analysis of variance and the Kruskal–Wallis test for independent samples. A significance level of *p* ≤ 0.05 was adopted.

### Apoptosis Assay (TUNEL)

A fluorometric DNA fragmentation detection kit (PromoCell, Heidelberg, Germany) was used to detect DNA ends, following the manufacturer’s instructions. Digital micrographs were acquired using a BZ8000 fluorescence microscope (Keyence, Neu-Isenburg, Germany).

## Results

**Figure 1:**
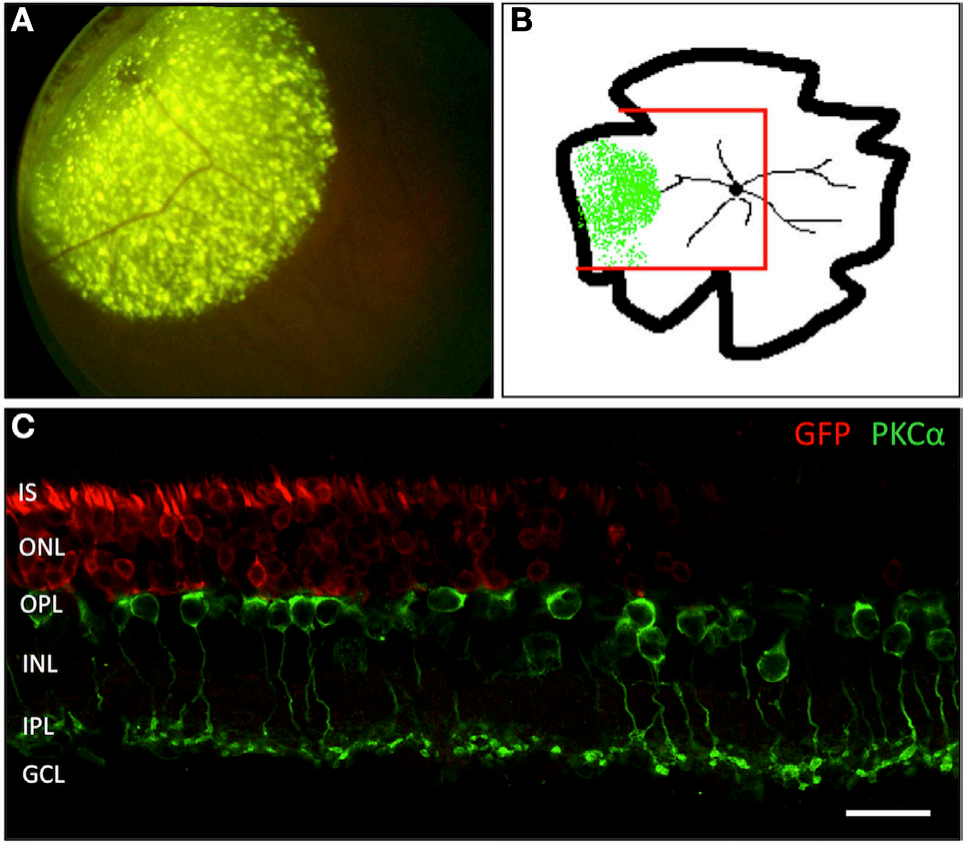
**Localization of the adeno-associated virus-transduced area by green fluorescent protein (GFP) expression**. **(A)** *In vivo* funduscopic image of a transduced rat retina 4 weeks after subretinal injection into the mid-peripheral retina. The strong, punctate fluorescence signal corresponds to the GFP expression in RPE cells. The weaker, homogenous fluorescence corresponds to the GFP expression in transduced PR cells. **(B)** Sketch of a flat-mounted retina. The area of GFP expression is marked green. The red lines indicate the portion prepared for vertical cryosections. **(C)** Vertical section through the area highlighted in panel **(B)** showing the transition zone between the GFP-transduced area and the non-transduced area lying toward the central portion of the retina. *n* = 7 transduced eyes. IS, inner segments of PR; ONL, outer nuclear layer; OPL, outer plexiform layer; INL, inner nuclear layer; IPL, inner plexiform layer; GCL, ganglion cell layer. Scale bar in panel **(C)**: 20 µm.

To visualize the transduced area, both *in vivo* funduscopy and fluorescence microscopy were carried out. The AAV2/5 vector used in this study is known to transduce with high-efficiency photoreceptors and RPE cells following subretinal injection. While we cannot assure 100% transduction efficacy inside the injected area, we do consider that almost all photoreceptor cells have been successfully targeted based on the images seen in Figure 1. *In vivo* funduscopy showed clearly visible GFP fluorescence restricted to the injection site. In retinal flat mounts, the GFP fluorescence was easily discernible using a fluorescence microscope with laser stimulation at 488 nm. In retinal vertical sections, transduced cells were labeled with GFP antibodies, showing a strong and homogenous signal in retinal photoreceptors (PR). The border of the transduced zone was clearly discernible, allowing comparison of retinal morphology within and outside the treated zone in the same eye (Figure 1).

### Morphological Structures Are Not Altered 1 and 12 Months after Subretinal Injection

**Figure 2:**
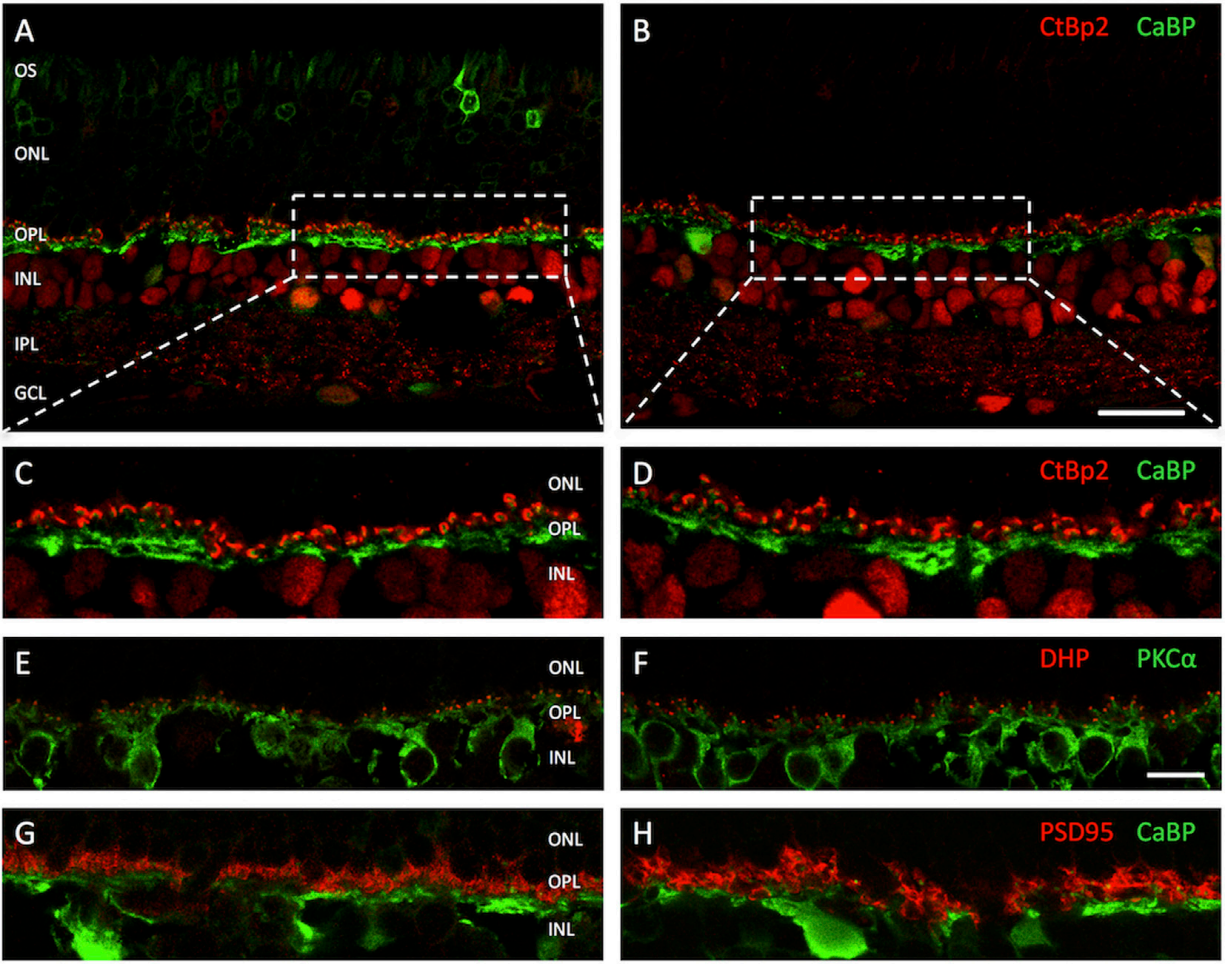
**Vertical cryosections of the retinas of adeno-associated virus (AAV)-transduced (left column) and untreated control animals (right column) with antibodies against different outer plexiform layer (OPL) structures**. **(A)** AAV-transduced area in treated animal. The GFP fluorescence in the photoreceptors is clearly discernible. **(B)** Untreated control. **(C,D)** Higher magnification images of the areas highlighted in panels **(A,B)** with antibodies against C-terminal-binding protein 2 (CtBP2) (red) staining photoreceptor synaptic ribbons and calcium-binding protein (CaBP) (green) marking horizontal cells. **(E,F)** OPL of treated (left) and untreated (right) retinas with antibodies for DHP (red) marking voltage-gated calcium channels on invaginating bipolar cell dendritic tips and PKCα (green) marking rod bipolar cells. **(G,H)** OPL of treated (left) and control (right) retinas with antibodies for PSD95 (red) and CaBP (green). Structure and arrangement as well as overall number of synaptic contacts between first and second order neurons do not appear to be altered in treated retinas. *n* = 5 AAV-transduced eyes and *n* = 5 untreated controls. Abbreviations: compare Figure 1. Scale bar in panel **(B)** [for panels **(A,B)**]: 20 µm; scale bar in panel **(F)** [for panels **(C–H)**]: 10 µm.

Antibodies against calcium binding protein (CaBP), CtBP2, dihydropyridine (DHP), protein kinase C (PKCα), and postsynaptic density protein (PSD95) were used to label structures of the OPL in vertical cryosections. With PSD95-immunolabeling PR synaptic terminals showed a homogenous distribution in two to five tightly packed rows (mostly representing rod spherules) immediately adjacent to the innermost cell layer of the outer nuclear layer (ONL) and above the OPL neuropil (Figures 2G,H). Similarly, with CtBP2 labeling, the synaptic ribbons showed a regular distribution within the OPL with no signals being dispersed into the adjacent ONL or inner nuclear layer (INL) (Figures 2A,B). In higher magnification micrographs, the horseshoe-like structure of the ribbon within rod spherules was clearly discernible (Figures 2C,D). Arrangement of rod spherules or photoreceptor terminals in general did not show anomalies in transduced retinas. The horseshoe structure of the synaptic ribbon was preserved and did not show shortening, fractioning, or clumping as has been described for animal models of retinal detachment or retinal degeneration. Particularly, no ectopic synapses were apparent in treated, sham-injected, and control animals, respectively.

PKCα immunolabeling showed a normal staining pattern for rod bipolar cells, their dendritic branches, contacting approximately 15–50 rod spherules each, being clearly distinguishable as fine strands stretching vertically through the OPL until just underneath the ONL (Figures 2E,F). As an indicator for functioning signal transmission from rods to rod bipolar cells DHP antibodies were used, highlighting clusters of voltage-gated calcium channels at the invaginating tips of bipolar cells (Figures 2E,F). With usually two tips of distinct bipolar cells present in each rod spherule synaptic invagination, giving it the typical triad arrangement, this results in a fluorescence pattern of two roughly circular and closely adjoined dots. Both, in treated retinas and controls, the DHP signal was directly mounted on the tips of rod bipolar dendritic processes and in close apposition to the synaptic ribbon in rod spherules. No loss or pruning of terminal dendritic branches in treated or sham-injected retinas was observed. Neuritic outgrowth or downregulation of postsynaptic receptor molecules could not be detected.

Green fluorescent protein expression within the PR remained stable even 12 months after treatment (data not shown). No alteration of OPL synaptic structures was observed with the markers used. Particularly, no PR terminal withdrawal or second-order neurite outgrowth were present in treated retinas. Overall retinal thickness appeared reduced in the older animals. However, this effect was likely due to age-associated degeneration or in any case light-induced damage, as it was equally observed in untreated controls.

### No Ultrastructural Modifications of the Photoreceptor Ribbon Synapses

**Figure 3:**
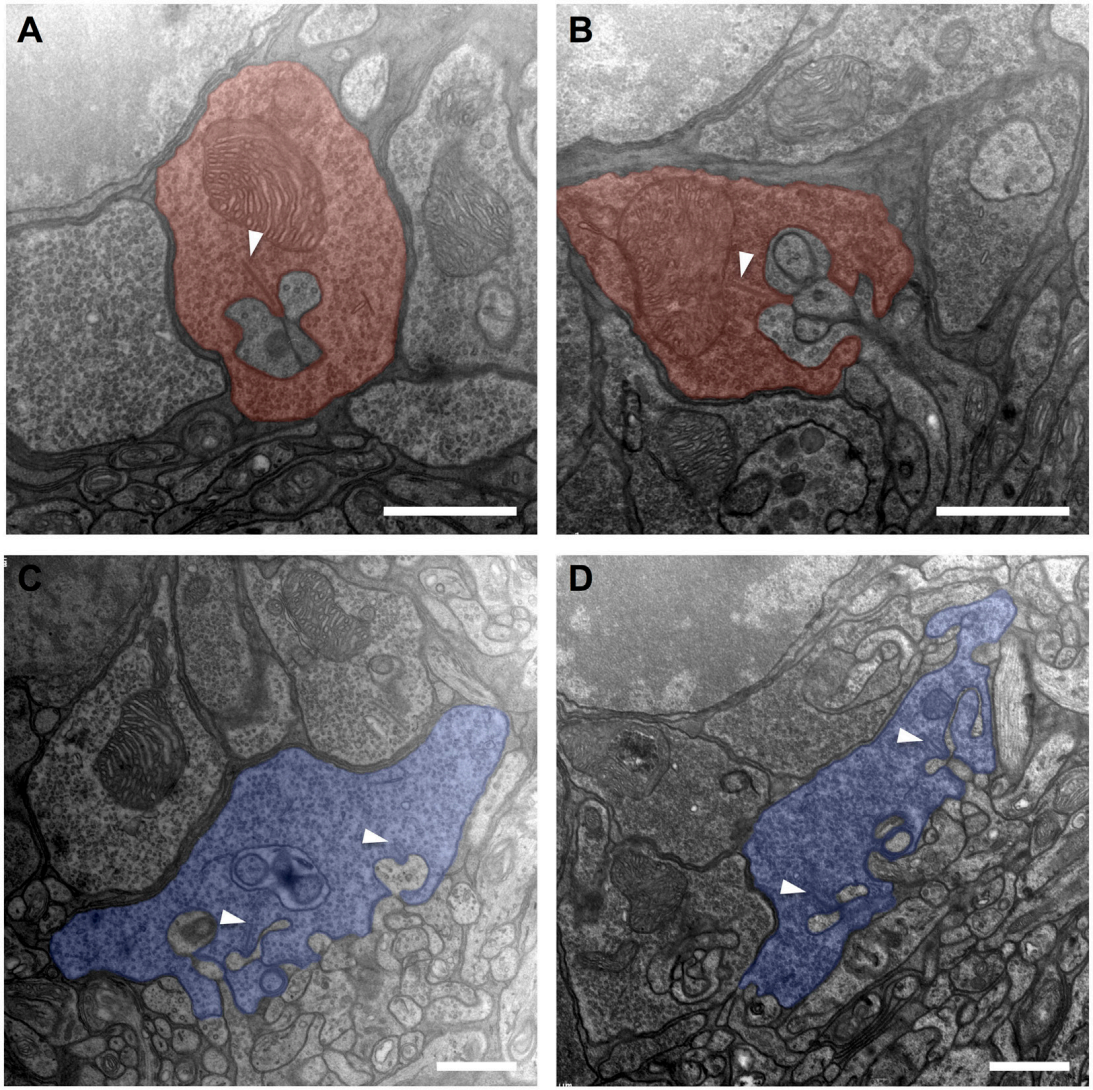
**Electron micrographs from the outer plexiform layer of adeno-associated virus (AAV)-transduced [left column: panels (A,C)] and untreated control [right column: panels (B,D)] retinas**. **(A,B)** Rod spherules (red) displaying a synaptic ribbon (arrowheads) opposing the invaginating dendrites of horizontal and bipolar cells. In conjunction, these three elements form what is called a synaptic triad. **(C,D)** Cone pedicles (blue) with invaginating and basal synaptic cell contacts. *n* = 2 AAV-transduced eyes and *n* = 2 untreated controls. Arrowheads: ribbon synapses. Scale bar in panels **(A–D)**: 1 µm.

Compared to untreated controls, the overall shape of rod terminals and their compact arrangement above the OPL neuropil within transduced areas appeared unchanged (Figures 3A,B). The cytoplasm was packed with synaptic vesicles, and the synapse with the second-order cells showed the typical triad structure with one clearly discernible ribbon and the associated invaginating processes of two horizontal cell axons as lateral elements and one to two bipolar cell dendrites in the center. The invaginations had a depth of approximately 1 µm, and the narrow hilus through which second-order neurites have to pass was clearly visible. Structural alterations as described after retinal detachment, such as fractioning or shortening of the synaptic ribbon, flat or missing invaginations of the basal cell membrane, or thickening of second order neurites along with disaggregation of the OPL neuropil, were not observed.

Similarly, cone pedicles in transduced areas had an unaltered overall aspect concerning shape and arrangement (Figures 3C,D). They showed an orthogonal diameter of approximately 5–7 µm, and their flat base was disrupted by multiple deep invaginations. Immediately adjoining the base of the cone pedicle lays the OPL neuropil with tightly packed processes of horizontal and bipolar cells. The fine postsynaptic processes, especially those of the horizontal cells, did not show any signs of swelling or hypertrophy compared to untreated controls. Within the cone pedicle, a number of intact ribbons were present, which were organized to typical triads with their postsynaptic partners. Basal cell contacts also did not show obvious discrepancies compared to healthy controls.

### Absolute Numbers of Ribbons Are Similar in Transduced and Non-Transduced Areas

**Figure 4:**
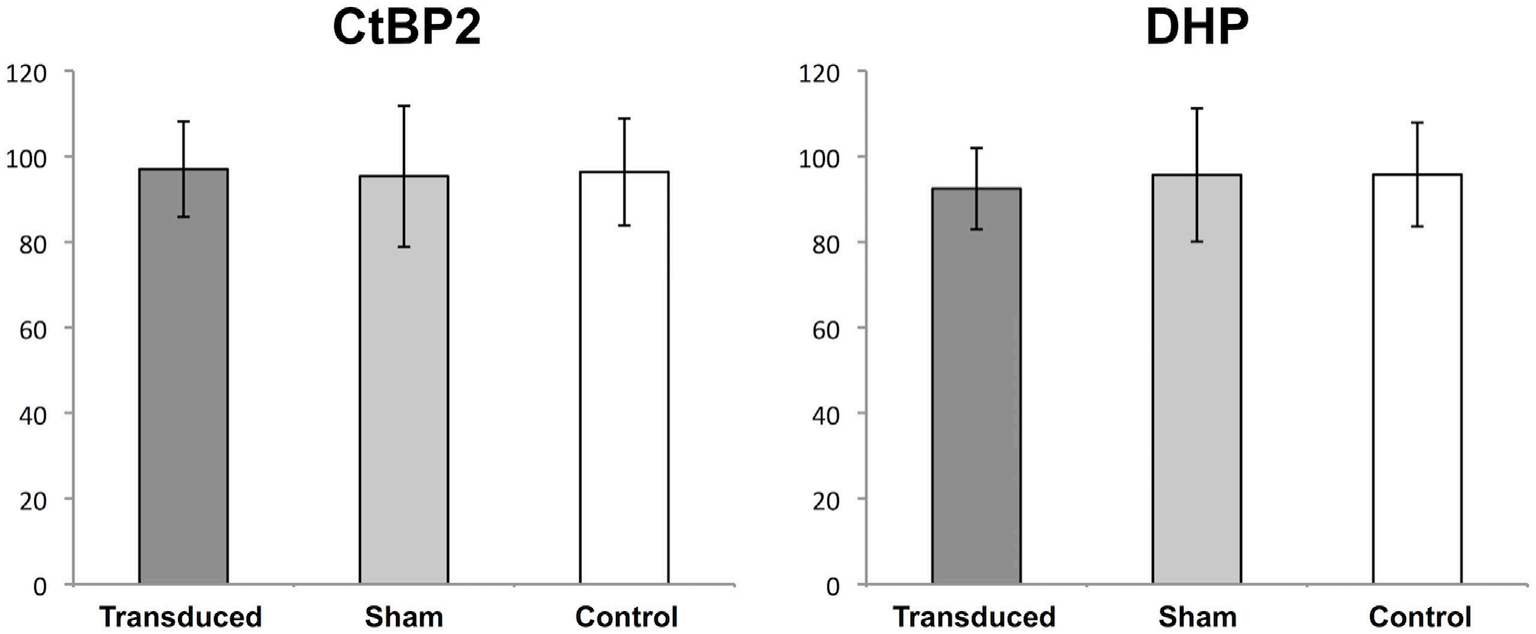
**Quantification of synapses in the outer plexiform layer (OPL)**. Boxplots showing the mean number (±1 SD) of counted signals in the OPL for synaptic ribbons [C-terminal-binding protein 2 (CtBP2)] and bipolar cell dendritic tips (DHP) in adeno-associated virus (AAV)-transduced areas (transduced), areas sham-injected with fluorescein (sham), and untreated controls (control). *n* = 3 for AAV-transduced and fluorescein-injected eyes, and *n* = 6 for untreated control eyes.

To examine the question of whether the transgene expression or the subretinal injection lead to a decrease in the number of synaptic interconnections within the OPL, the eyes of six unilaterally injected animals were studied 1 month after treatment (Figure 4). In this experimental setup, no statistically significant differences in the number of CtBP2- and DHP-positive signals were observed in the OPL of GFP-transduced, fluorescein-injected, and untreated eyes, respectively. In GFP-transduced retinas 97 (SD ± 11.1) CtBP2-positive synaptic ribbons were counted as opposed to 95.3 (±16.5) in the sham-injected retinas and 96.3 (±12.5) in the untreated controls (*p* = 0.966 and *p* = 0.816 for ANOVA and Kruskal–Wallis, respectively). Accordingly, 92.4 (±9.5) DHP-positive postsynaptic clusters were counted in GFP-transduced retinas as opposed to 95.8 (±12.1) in the sham-injected and 95.7 (±15.6) in the untreated control retinas (*p* = 0.810 and *p* = 0.645 for ANOVA and Kruskal–Wallis, respectively).

### Müller Cell Reaction

**Figure 5:**
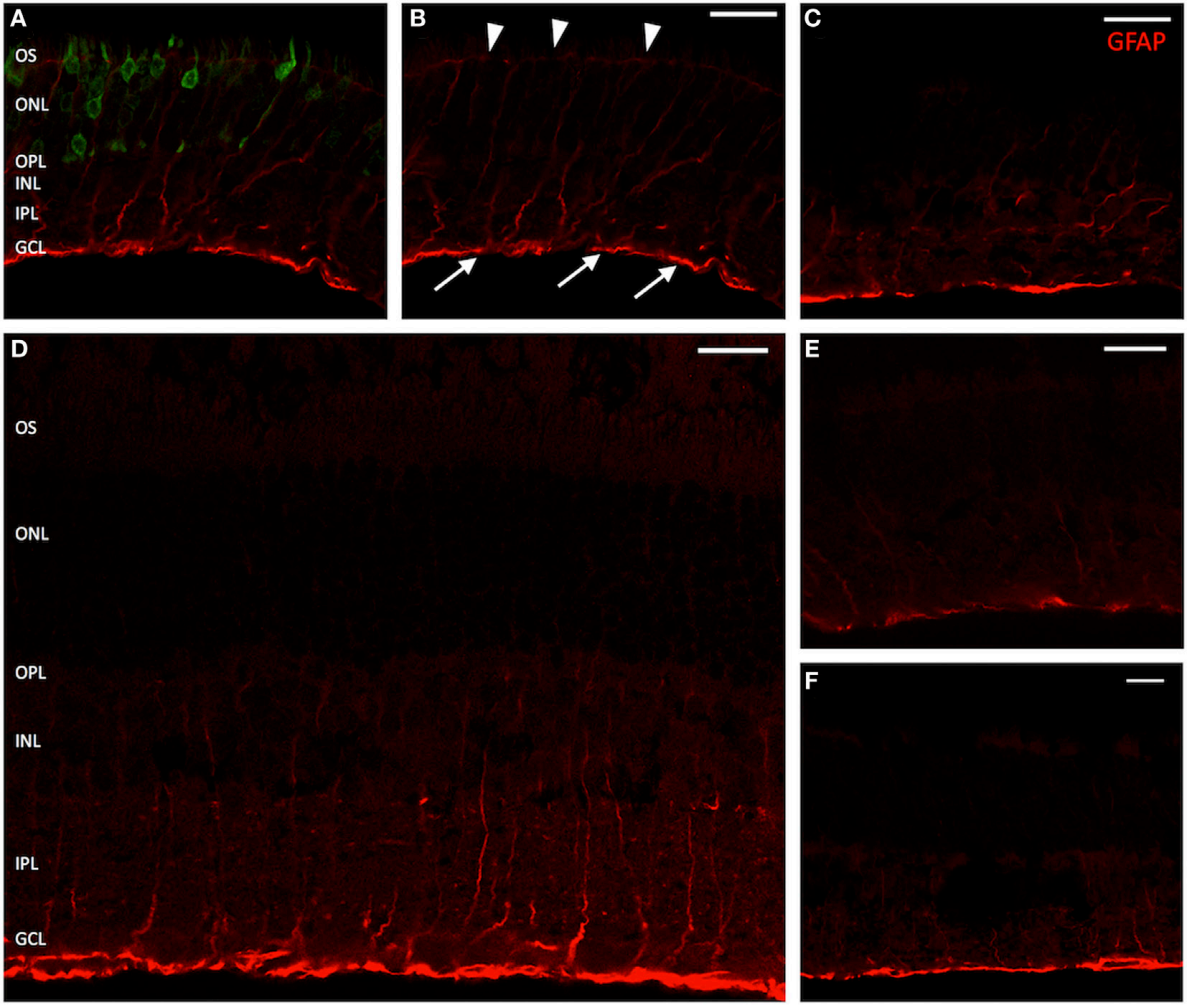
**Vertical sections of rat retinas with labeling for glial fibrillary acidic protein (GFAP) (Müller cells)**. **(A)** Green fluorescent protein (GFP)-transduced area. The GFP fluorescence within photoreceptors is clearly discernible. **(B)** Red channel from panel **(A)**. Arrow heads mark the outer limiting membrane; arrows point out the inner limiting membrane. **(D)** Section through a retina sham injected with fluorescein. **(C,E)** Healthy controls, peripheral retina. **(F)** Healthy control, central retina. The GFAP signal is clearly elevated in treated and sham-injected areas, extending far into the inner nuclear layer (INL) and sometimes staining the entire Müller cell body. *n* = 3 for adeno-associated virus-transduced and fluorescein-injected eyes, and *n* = 6 for untreated control eyes. Abbreviations: compare Figure 1. Scale bar in all panels: 20 µm.

On immunostaining for glial fibrillary acidic protein (GFAP), untreated eyes displayed a fluorescence pattern limited to the nerve fiber or ganglion cell layer (GCL) (Figures 5C,E,F). This corresponds to the distributional pattern of retinal astrocytes, which are regularly GFAP-positive and are involved both in the formation of the blood–retinal barrier and the inner limiting membrane (ILM), as well as that of Müller cell endfeet, in which GFAP is also weakly expressed. In GFP-transduced animals, a distinct fluorescence pattern of radially arranged fibers was observed within the area of GFP expression (Figures 5A,B), which extended throughout the entire retina from the inner to the outer limiting membrane and corresponds to the typical fluorescence pattern of Müller cells. Similarly, eyes that only received a sham injection with fluorescein displayed fibrillary “sprouts,” reaching from the ILM far into the INL (Figure 5D), which was well consistent with the fluorescence pattern seen in Müller cells after traumatic manipulation.

An increase of Müller cell processes in the regions of the inner or outer limiting membrane and particularly outgrowths of GFAP-positive fibrils into the subretinal or vitreal space were not observed.

### No Sign of Increased Apoptosis in the Transduced Area

Two methods were used to assess the apoptotic status of examined retinas: immunostaining for apoptosis-inducing factor (AIF) and a TUNEL assay to detect DNA double-strand breaks.

**Figure 6:**
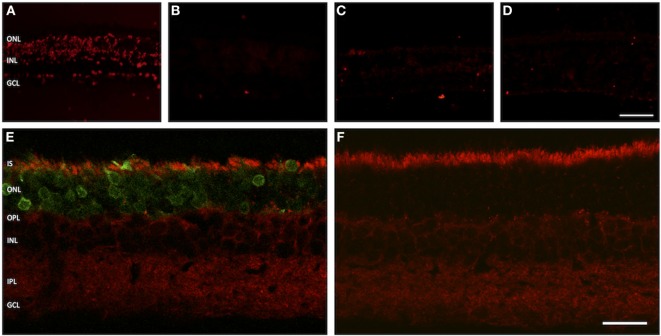
**Assessment of apoptosis in treated versus untreated retinas**. **(A–D)** TUNEL assay. **(A)** Positive control. **(B)** Negative control. **(C)** Adeno-associated virus (AAV)-transduced retina. **(D)** Retina from untreated eye. **(E,F)** Vertical cryosections with staining for apoptosis-inducing factor (AIF). **(E)** AAV-transduced retina. The green fluorescent protein fluorescence in the photoreceptors is easily discernible. **(F)** Untreated control retina. The AIF signal is evenly distributed in the inner segments of photoreceptor, showing no sign of dislocation into the cell bodies. *n* = 3 for AAV-transduced and fluorescein-injected eyes, and *n* = 6 for untreated control eyes. Abbreviations: compare Figure 1. Scale bar in panel **(D)** [for panels **(A–D)**]: 50 µm. Scale bar in panel **(F)** [for panels **(E,F)**]: 20 µm.

Apoptosis-inducing factor is a flavoprotein expressed by eukaryotic cells that plays a key role in initiating programmed cell death. It is usually located within the inter-membranous gaps of mitochondria. In apoptotic cells, a dislocation of AIF into the cytosol and the nucleus is observed. Immunolabeling of treated and sham-injected rat retinas with AIF antibodies leads to a strong fluorescence signal within the PR inner segments, where most of the cells mitochondria are located. A dislocation of the fluorescence signal into the cell body was not observed (Figures 6E,F).

In the TUNEL assay, DNA double-strand breaks are made visible by specific binding of free 3′-OH ends of DNA fragments. In our experimental setup, neither GFP-transduced nor sham-injected retinas showed an increased number of apoptotic nuclei as compared to retinal regions from outside the injection bleb or healthy controls (Figures 6A–D).

## Discussion

In this study, we show that subretinal delivery of AAV vectors does not cause significant alterations to structures in the OPL in terms of qualitative features, such as increased sprouting of bipolar cells or synaptic ribbon structures, or quantitative features, such as the absolute number of synaptic terminals. This intervention also does not cause increased rates of photoreceptor apoptosis.

When looking at AAV gene transfer into the retina by subretinal delivery route, several issues have to be taken into account when analyzing the effects of such an intervention on the morphology of the photoreceptor cells. Subretinal delivery causes a transient retinal detachment, the intracellular presence of the viral vector potentially induces cellular stress, and the transgene can prevent the transduced cells from functioning normally. While the latter point is directly related to the amount of GFP produced in photoreceptor cells, it was shown that even high intracellular concentrations of GFP did not have toxic effects (35, 36). Likewise, even though it has been shown that AAV vector particles can persist in photoreceptors following subretinal injection for a long time (37), any toxicity related to the viral vector itself has not been reported so far.

By contrast, it is known that AAV-mediated gene delivery by subretinal injection leads to a transient retinal detachment, which in most cases resolves spontaneously within 24–48 h, by which time photoreceptors and retinal pigment epithelial cells have been transduced by the viral vector. The retinal detachment itself can lead to a number of distinct changes in retinal morphology, ultrastructure, and protein expression of retinal cells. The changes that detached retinas undergo follow a reproducible pattern and have been described in great detail (29). Part of these processes are reversible and seem to have in fact a protective function by downregulating cell metabolism and thereby enabling cell survival in detached retinal regions (38).

The volume that is introduced into the subretinal space determines in large part the area being detached, which means that larger volumes generally detach a larger retinal zone. This is associated with shear stress to the retina, representing a potentially negative factor concerning the morphological integrity. In our study, we injected 1.5 µL vector solution, thereby resulting in the detachment of about 20% of the retinal zone. No apparent morphological changes with regard to this stress factor were observed. Since we did not detach a larger area, the physical stress to the detached healthy retina was probably at a lower level as compared to the stress induced when larger parts of the retina would be detached. However, in clinical trials, retinal detachments are also limited to defined areas and do not include more than 30% of the retina being detached at once, making our setup relevant in this regard (14–17). However, one limitation remains the fact that we were studying the effects in a healthy retina, while the consequences of physical stress during the detachment might be more important in retinal tissue undergoing neuroretinal degeneration. This question is now subject to ongoing investigations.

Synaptic sprouting has also been observed in animal models of experimental retinal detachment. However, the level of sprouting appears to depend on the duration of the detachment and is also in part reversible (29, 30, 38). Even in retinas that had been detached for several days, a “restructuring” of the OPL was observed with most ectopic synapses disappearing and ONL thickness increasing within a couple of weeks after reattachment (29, 38).

In our study, we did not observe increased rates of sprouting when looking at the OPL of retinas 1 or 12 months following subretinal AAV gene delivery. It is very likely that any potential transient changes that might have occurred shortly after the delivery were reversed and did not have a pronounced effect on the structure of the photoreceptor terminals. Likewise, the absolute number of ribbon synapses was not altered in retinas transduced by AAV vectors or retinas injected with fluorescein alone, indicating that the AAV delivery itself is not harmful to the retinal structure. The presence of AAV particles in confined compartments within the photoreceptor terminals, which was shown in a previous study (37), and which might also be the case in the terminals of transduced photoreceptors analyzed in this study (although not detectable on EM images), also does not seem to have an effect on the synaptic architecture.

Müller cells seem to react very rapidly to an experimental retinal detachment by changing their expression profile, usually leading to a pronounced upregulation of GFAP expression. Similarly, we also observed an increased expression of GFAP in the transduced retinae, seen as fibers in parallel formation stretching from the ILM and the GCL into the outer retinal layers, and in some cases all the way to the outer limiting membrane, corresponding to the typical shape and arrangement of Müller cells (39), and indicating a similar response of the Müller cells to subretinal delivery of AAV. Since the same reaction was found both, in animals that received subretinal gene transfer and in animals that were sham injected with fluorescein, we conclude that the upregulation of GFAP in Müller cells seen in our study is a consequence of the transient retinal detachment itself and not related to the gene delivery or the presence of viral particles in the retina.

Increased rates of apoptosis of photoreceptor cells were found in experimental retinal detachment studies (40). Apoptotic activity was strongest between 24 and 72 h after detachment, at which time approximately 20% of photoreceptors were lost (38, 40). However, even in long-term detachment (up to 450 days in the animal model), about half of the photoreceptor population was preserved (41). In our study, we did not find an increase of apoptotic nuclei in GFP-transduced areas as compared to sham-injected retinas and healthy controls. Furthermore, AIF was not delocalized from the PR IS into the cytosol and the nucleus. Taking into account the normal overall appearance of the retinal tissue by light microscopy and comparable retinal layer thickness, we conclude that there is no increase of apoptotic cell death in GFP-transduced and sham-injected retinas, respectively. Likewise, the unchanged level of apoptotic cells suggests that the presence of AAV following the subretinal injection does not represent a toxic insult to the photoreceptor cells, an observation that goes in line with the absence of any toxic signs in many preclinical and clinical trials of AAV-mediated gene delivery.

## Conclusion

Adeno-associated virus-mediated transgene delivery *via* subretinal injection and expression of GFP as a transgene in photoreceptors did neither inflict significant changes on OPL morphology in healthy rats nor did they lead to increased rates of apoptotic cell death. Therefore, this study adds further evidence to the general assumption that AAV-mediated gene transfer is a safe and efficient way for transgene delivery to outer retinal cells.

## Author Contributions

KS designed the project, supervised the people performing all experiments, wrote the paper, and revised the paper. BG performed the experiments and wrote the paper. DK, AM-M, and CI provided material, performed experiments, and revised the paper. BL revised the paper. SH supervised the people performing the experiments and revised the paper.

## Conflict of Interest Statement

The authors declare that the research was conducted in the absence of any commercial or financial relationships that could be construed as a potential conflict of interest.
